# Gut biomolecules (I-FABP, TFF3 and lipocalin-2) are associated with linear growth and biomarkers of environmental enteric dysfunction (EED) in Bangladeshi children

**DOI:** 10.1038/s41598-022-18141-8

**Published:** 2022-08-16

**Authors:** Md. Mehedi Hasan, Md. Amran Gazi, Subhasish Das, Shah Mohammad Fahim, Farzana Hossaini, Ar-Rafi Khan, Jafrin Ferdous, Md. Ashraful Alam, Mustafa Mahfuz, Tahmeed Ahmed

**Affiliations:** 1 Nutrition and Clinical Services Division, International Centre for Diarrhoeal Disease Research, Bangladesh (icddr,b), Dhaka, 1212 Bangladesh; 2 Faculty of Medicine and Life Sciences, University of Tampere, Tampere, Finland; 3 James P. Grant School of Public Health, BRAC University, Dhaka, Bangladesh; 4 Department of Global Health, University of Washington, Seattle, WA USA

**Keywords:** Diagnostic markers, Predictive markers

## Abstract

In the current world, a major challenge to diagnose environmental enteric dysfunction (EED) is the lack of validated non-invasive biomarkers. Intestine derived molecules, including intestinal fatty acid binding protein (I-FABP), trefoil factor-3 (TFF3), lactoferrin, lipocalin-2 (LCN2), and mucin-2, have been reported as indicators of intestinal inflammation and gut health. Therefore, we aimed to investigate the levels of these bio-molecules as biomarkers of EED among under-2 children in Bangladesh. A total of 140 children were recruited in a case–control design. All the biomarkers were measured by ELISA. Spearman’s rank correlation was performed to see the correlation between the biomarkers and the EED score. Moreover, multivariable linear regression was performed to investigate the association of biomarkers with length-for-age z-score (LAZ). TFF3 correlates positively with myeloperoxidase (r = 0.26, p < 0.05) and EED score (r = 0.17, p < 0.05). Likewise, LCN2 correlates positively with myeloperoxidase (r = 0.37, p < 0.05), neopterin (r = 0.33, p < 0.05) and EED score (r = 0.31, p < 0.05). Moreover, multivariable linear regression revealed a negative association of I-FABP with LAZ of the study participants. Our results imply that TFF3 and LCN2 might be promising biomarkers to diagnose intestinal inflammation and EED, while I-FABP is negatively associated with linear growth of Bangladeshi children.

## Introduction

Environmental enteric dysfunction (EED) is a sub-clinical gut inflammation which is characterized by crypt hyperplasia, villous atrophy, and infiltration of lymphocytes in lamina propria^1^. It has been found to be associated with stunted growth among children in developing countries^1^. Moreover, EED affects almost 40% of children under the age of 5 years in low and middle-income countries and causes growth retardation or stunting^2^. Reduced vaccine responsiveness and altered gut microbiota are the most important consequences of EED. According to the studies, 80% of infants become EED positive within 12 weeks of birth, and 50% of under-5 children are stunted in the slum area^3–6^. Therefore, our current understanding reflects EED as a driver of stunted growth of children in resource-poor settings. Thus, there arises a demand to concentrate on preventing and treating EED successfully to reduce the overall burden of stunting. However, there is currently a paucity of non-invasive biomarkers for EED, which presents a major challenge in diagnosing EED as well as in evaluating the treatment and prevention strategies for EED.

To date, intestinal biopsy has been used to diagnose EED^1^. However, collection of small intestinal biopsy samples is technically infeasible in young children. Studies have included non-invasive biomarkers of intestinal damage, repair, and permeability as well as intestinal inflammation to diagnose EED^1^. However, none of the studies have correlated these non-invasive biomarkers with the composite EED score that has been proposed based on neopterin (NEO), myeloperoxidase (MPO), and alpha-1 anti-trypsin (A1AT) to diagnose EED and the findings were published earlier^7^. Several studies have also used this composite score for the better prediction of EED and intestinal inflammation^8–13^. In addition, EED score has also been found to be correlated with LCN2 in our previously published cross-sectional study in Bangladesh^14^. Though MPO, NEO, and A1AT are non-invasive biomarkers to generate a composite EED score, these biomarkers only predict intestinal inflammation and intestinal permeability to diagnose EED. Whereas EED is a complex phenomenon that is characterized by different features published earlier^1^. So, more precise candidate biomarkers are needed for the proper diagnosis through prediction of different features of EED. To the best of our knowledge, some intestine derived molecules such as intestinal fatty acid binding protein (I-FABP), trefoil factor-3 (TFF3), faecal lactoferrin, faecal lipocalin-2 (LCN2), and mucin 2 (MUC2) have been reported as indicators of intestinal inflammation and intestinal health in various studies^1,15–18^. I-FABP is an intracellular epithelial protein that has been reported as an indicator of the severity of intestinal damage in different age groups^1^. Lactoferrin is a major component of neutrophilic granulocytes. It is known as a sensitive and specific marker of inflammation among patients with inflammatory bowel disease (IBD)^15^. TFF-3 has been found to play an important role in wound healing and epithelial regeneration. It has also been reported as a powerful diagnostic tool to determine gut injury in animal model^16^. LCN2 is a glycoprotein that is secreted by neutrophils. Expression of LCN2 is upregulated in tissue damaging conditions including burn injury, ulcerative colitis, and infection where free radicals are produced^17^. MUC2 is the main component of the intestinal mucus layer, secreted by the goblet cells. And it’s deficiency leads to inflammation of the colon and growth arrest in animal model^18^. Since it is a main component of the mucus layer of intestine, thus lower levels of MUC2 in stool may arise for the reduction of the mucus layer. But no study has been conducted to investigate these bio-molecules as biomarkers of EED as well as their levels in cases of severe stunting who have the higher possibility of having EED. Therefore, an attempt was made to investigate these biomolecules as biomarker signatures of EED. So, we intended to investigate the levels of these bio-molecules in children with severely stunted and healthy controls in Bangladesh in the context of EED.

## Methods

### Study design, participants, study setting

We employed a case–control design where severely stunted (LAZ <  − 3)^19^ children were cases (n = 76) and healthy children (LAZ >  − 1, not suffering from any chronic disease such as cerebral palsy, TB, trisomy-21, congenital heart disease, chronic liver or renal disease, cleft lip/palate, epilepsy etc.) were recruited as controls (n = 64). Biological samples of the cases were collected from the “Bangladesh environmental enteric dysfunction (BEED)” study, and the detailed protocol of this study has been published elsewhere^2^, whereas the control participants were recruited from Bauniabad, a slum of Dhaka, which is inhabited by middle-class and poor families, and their residential and sanitary conditions are typically poor.

### Data collection, sample collection, and laboratory analyses

Anthropometric, socio-economic, and biomedical data were collected by trained field staff. Weight-for-length z-score (WLZ), weight-for-age z-score (WAZ), length-for-age z-score (LAZ), head circumference, and mid-upper arm circumference (MUAC) were calculated to assess the nutritional status of the participants. 4 mL of venous blood and 6 g of stool were collected from each of the participants. All the samples were transported to the laboratory under adequate cold chain maintenance. Whole blood was centrifuged at 3000×*g* for 10 min to collect plasma. Aliquots of plasma and stool samples were stored in − 80 °C freezers until biomarker analysis. Biomarkers such as I-FABP (R&D system, USA; detection limit: 15.6 pg/mL to 1000 pg/mL), TFF3 (R&D system, USA; detection limit: 39 pg/mL to 2500 pg/mL), RBP4 (R&D system, USA; detection limit: 1.6 ng/mL to 100 ng/mL), CRP (Immundiagnostik, Germany; detection limit: 1.9 ng/mL to 150 ng/mL) and ferritin (ORGENTEC, Germany, detection limit: 15 ng/mL to 1500 ng/mL) were measured in plasma as well as faecal biomarkers including LCN2 (R&D system, USA; detection limit: 0.156 ng/mL to 10 ng/mL), MUC2 (MyBioSource, USA; detection limit: 0.312 ng/mL to 20 ng/mL), Lactoferrin (TECHLAB, USA; detection limit: 6.25 ng/mL to 100 ng/mL), MPO (Immundiagnostik, Germany; detection limit: 1.9 ng/mL to 30 ng/mL), NEO (GenWay Biotech, USA; detection limit: 1.35 nmol/L to 111 nmol/L), and A1AT (Biovendor, USA; detection limit: 3.3 ng/mL to 90 ng/mL) were measured by using commercially available ELISA kits. In our laboratory analyses, plasma and stool samples were diluted according to the kit manual to keep the result within the range of concentration of the standard. Finally, the obtained concentration of the biomarker in sample was multiplied with the dilution factor to find out the final result. All the laboratory assays were done at icddr,b.

### Sample size calculation

The formula that we used for calculating sample size is—[n = {(r + 1)/r} × {SD^2^ × (Z_*β*_ + *Z*_*α*_*/*2)^2^*}*/d^2^, where r = ratio of case to control, SD = standard deviation, d = expected mean difference between case and control, Z_*β*_ = 0.84, *Z*_*α*_*/*2 = 1.96]^20^. We used data from a published article where the authors reported the mean difference (d) and standard deviation (SD) for I-FABP to be 234.3 pg/mL and 434, respectively, comparing healthy control with Crohn’s disease (CD) patients^21^. We considered 80% power and the level of significance as 0.05. With an equal allocation (1:1) between cases and controls, total sample size estimated using this formula was 54 for each study arm. However, we also calculated sample size using the same equation for other biomarkers such as MUC2, LCN2, lactoferrin, and TTF-3, but sample sizes for all biomarkers were less than 54 in each group. Hence, we decided to use the larger sample size of 108 children (54 cases and 54 controls) using the data for I-FABP.

### EED score

A principal component analysis (PCA) was performed by Kosek et al.^7^ to create a composite score of environmental enteric dysfunction (EED) based on MPO, NEO, and A1AT using the following equation where MPO, NEO, A1AT categories were defined as 0 (≤ 25th percentile), 1(25–75th percentile) and 2 (≥ 75th percentile). The range of the EED score is 0 to 10.

$${\text{EED score = 2 }} \times {\text{ (MPO category) + 2 }} \times {\text{ (A1AT category) + 1 }} \times {\text{ (NEO category)}}{.}$$

### Conceptual framework for selecting newly proposed biomolecules as biomarkers of EED

**Figure 1:**
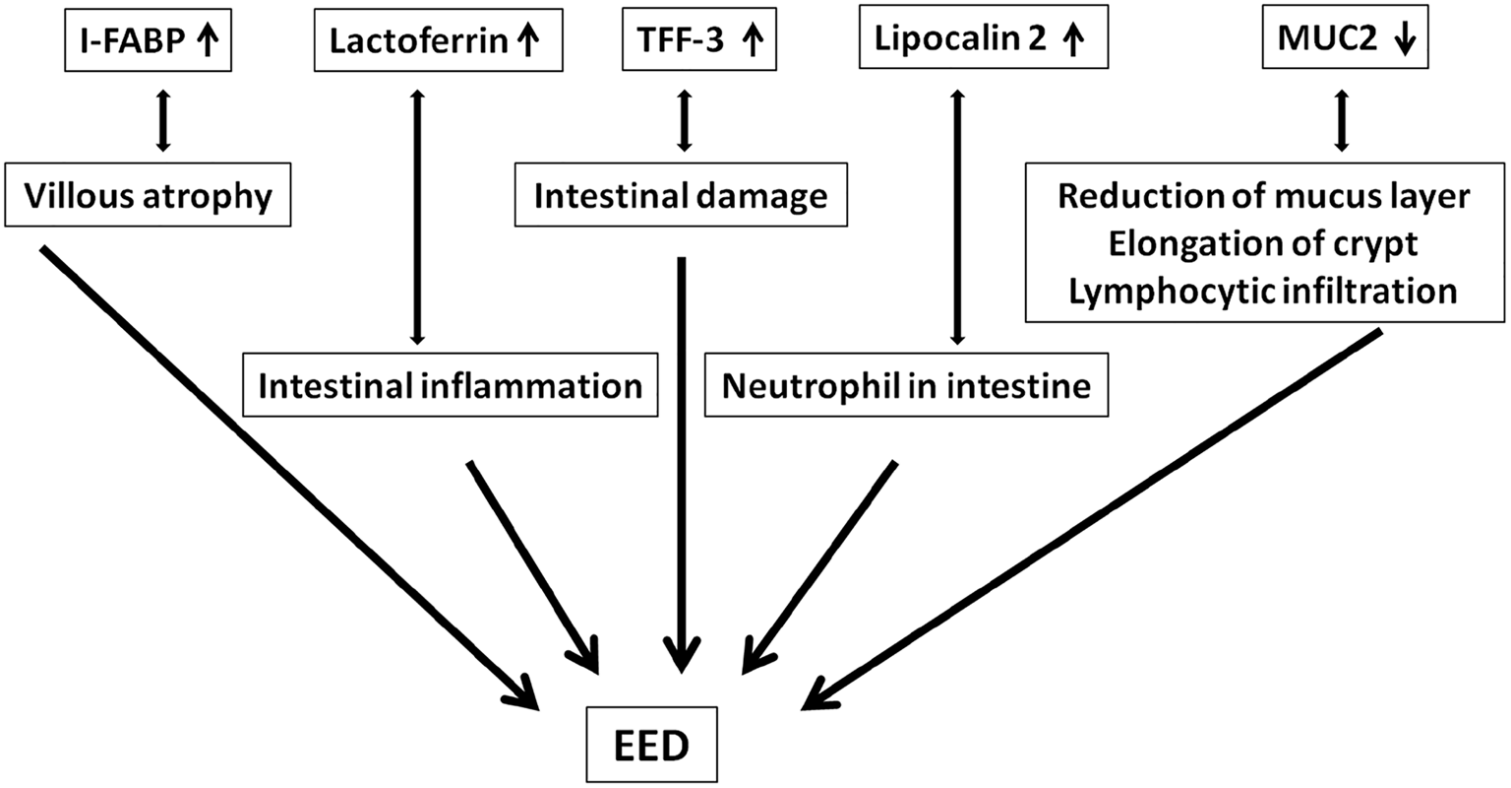
Increased plasma I-FABP is an indication of villous atrophy^35^, increased faecal lactoferrin is an indication of intestinal inflammation^15^, increased plasma TFF-3 suggests intestinal damage^16^ and increased faecal lipocalin 2 represents the presence of neutrophil in lamina propria^17^. On the other hand, lower levels of faecal MUC2 describe the reduction of mucus layer, elongation of crypt and lymphocytic infiltration^18^. Overall, all these representations collectively describe the different dimensions of EED and related pathogenesis^1^. In the same way, this entire bio-molecules panel can indicate the presence of EED.

Figure 1 represents the conceptual framework to select these biomolecules such as I-FABP, lactoferrin, TFF-3, LCN2, and MUC2 to investigate as biomarkers of EED.

### Data analysis

The Mann–Whitney U test, student’s t-test, and Chi-square test were done to compare the characteristics of cases and controls. The Mann–Whitney U test was used for the variables showing skewed distributions, whereas the variables following a normal distribution were analysed by the student’s t-test. A Chi-square test was performed to analyse the categorical variables, which were presented as frequencies with percentages. Spearman’s correlation was used to measure the correlation between the biomarkers and the EED score. Multivariable linear regression was done to measure the associations of different variables with LAZ of the study participants, where LAZ was the outcome variable. The model was adjusted for age, sex, nutritional status, and socioeconomic status of the participants^17,22^. Additionally, the variables that showed p-value < 0.2 in bivariate analysis were included in the multivariable model. A probability value (p-value) < 0.05 was considered as statistically significant. All data analyses were done using STATA version −13.

### Ethics statement

The study protocol (PR-20025) was approved by the Institutional review board (IRB) of the International Centre for Diarrhoeal Disease Research, Bangladesh (icddr,b). Informed written consent was taken from the mothers of the study participants after describing the study.

### Statement of confirmation

All methods were carried out in accordance with relevant guidelines and regulations.

## Results

### Anthropometric and socioeconomic characteristics of the study participants

Table 1 shows the baseline characteristics of the study participants. In this study, the male and female ratio was almost similar in healthy cohort. However, male participants were higher compared to females in severely stunted group. The median (Q1, Q3) ages of the healthy and severely stunted children were 15.18 (13.36, 17.97) months and 14.90 (12.75, 16.8) months, respectively. Anthropometric indices such as weight, length, MUAC, head circumference, length-for-age z-score (LAZ), weight-for-age z-score (WAZ), and weight-for-length z-score (WLZ) were significantly lower in severely stunted children compared to their healthy counterparts. Moreover, maternal height, maternal education, and WAMI index were also significantly lower in severely stunted children. Family income was higher in healthy children, but not statistically significant.

**Table 1:** Anthropometric and socioeconomic characteristics of the study participants.

	Overall (n = 140)	Healthy control (n = 64)	Severe stunted (n = 76)	p-value
**n (%)**				
Gender				
Male	75 (53.57)	33 (51.56)	42 (55.26)	0.01
Female	65 (46.43)	31 (48.44)	34 (44.74)	
**Median (Q1, Q3)**				
Child age (month)	15.07 (13.10, 16.83)	15.18 (13.36, 17.95)	14.90 (12.75, 16.38)	0.06
Family income in USD (USD)	165.09 (119.21	176.88 (129.71, 235.84)	152.19 (119.21, 238.47)	0.31
**Mean (SD)**				
Weight in kg	8.47 (1.61)	9.97 (0.93)	7.18 (0.68)	0.0001
Length in cm	72.66 (5.49)	77.89 (3.09)	68.26 (2.22)	0.0001
MUAC in cm	13.95 (1.32)	15.14 (0.79)	12.94 (0.69)	0.0001
Head circumference in cm	44.16 (1.08)	45.43 (1.28)	43.08 (1.44)	0.0001
Length for age z score	− 2.17 (1.81)	− 0.30 (0.59)	− 3.74 (0.55)	0.0001
Weight for age z score	− 1.57 (1.53)	− 0.08 (0.64)	− 2.84 (0.69)	0.0001
Weight for length z score	− 0.62 (1.02)	0.07 (0.70)	− 1.21 (0.87)	0.0001
Maternal height in cm	148.79 (5.38)	150.65 (4.79)	147.18 (5.37)	0.0001
Maternal education (years)	6.14 (2.62)	6.78 (2.75)	5.45 (2.29)	0.003
WAMI	0.54 (0.11)	0.56 (0.12)	0.53 (0.10)	0.07

### Levels of biochemical parameters of the study participants

In our study, plasma biomarkers including I-FABP and C-reactive protein (CRP) as well as faecal biomarkers such as LCN2, MPO, and NEO were significantly higher in severely stunted children compared to healthy children. Moreover, TFF3 and ferritin were higher in severely stunted children, but the differences between the study groups were not statistically significant. On the other hand, RBP4, lactoferrin, MUC2 and A1AT were higher in healthy children. In addition to that, EED score is significantly higher in severely stunted children compared to healthy controls. Table 2 shows the levels of biochemical parameters of the study participants.

**Table 2:** Levels of plasma and faecal biomarkers of the study participants.

	Overall (n = 140)	Healthy control (n = 64)	Severe stunted (n = 76)	p-value
**Plasma biomarkers: median (Q1, Q3)**				
I-FABP (ng/mL)	1.63 (1.15, 2.44)	1.41 (1.08, 2.36)	1.94 (1.23, 2.76)	**0.03**
TFF3 (ng/mL)	17.24 (13.52, 24.35)	16.23 (12.73, 23.92)	17.60 (13.93, 25.73)	0.29
RBP4 (mg/L)	19.17 (15.35, 22.93)	19.47 (15.88, 22.62)	18.80 (14.81, 23.82)	0.83
CRP (mg/L)	0.56 (0.14, 2.43)	0.21 (0.07, 0.69)	1.75 (0.47, 5.22)	**0.0001**
Ferritin (ng/mL)	9.46 (6.18, 17.41)	8.49 (6.35, 14.42)	9.96 (6, 20.94)	0.37
**Faecal biomarkers: median (Q1, Q3)**				
LCN2 (µg/mL)	9.90 (3.77, 24.27)	8.34 (3.36, 15.54)	16.35 (4.55, 38.11)	**0.009**
Lactoferrin (ng/mL)	89.24 (30.75, 148.02)	103.78 (52.19, 165.30)	71.06 (22.47, 146.63)	0.06
MUC2 (ng/mL)	1.59 (1.17, 3.17)	1.71 (1.13, 3.04)	1.55 (1.20, 3.17)	0.91
MPO (ng/mL)	1983 (1073.5, 4382)	1317.75 (662.5, 3042.5)	3134 (1453.25, 7329.75)	**0.0001**
NEO (nmol/L)	801 (358, 1869)	571 (244, 850)	1394.5 (650, 2676)	**0.0001**
AAT (mg/g)	0.49 (0.23, 0.76)	0.53 (0.34, 0.80)	0.41 (0.11, 0.74)	**0.003**
**Score: median (Q1, Q3)**				
EED score		4 (3, 6)	5 (4, 8)	**0.03**

Significant values are in bold.

### Correlation of these newly proposed biomolecules with biomarkers of EED and EED score

A significant positive correlation was found between TFF3 and MPO (r = 0.26, p = 0.0025). TFF3 also positively correlates with the EED score (r = 0.17, p = 0.04). Likewise, LCN2 significantly and positively correlates with MPO (r = 0.37, p = 0.001), NEO (r = 0.33, p = 0.0001), and EED score (r = 0.31, p = 0.0003). Lactoferrin significantly and positively correlates with A1AT (r = 0.21, p = 0.01). However, MUC2 significantly and negatively correlates with MPO (r =  − 0.18, p = 0.03). In addition, I-FABP correlates with none of the biomarkers and the EED score. Table 3 illustrates the correlations among different variables.

**Table 3:** Correlation among different biomarkers.

Variables	I-FABP	TFF3	LCN2	Lactoferrin	MUC2	MPO	NEO	A1AT	EED score
I-FABP	1.00								
TFF3	0.21	1.00							
LCN2	− 0.01	0.006	1.00						
Lactoferrin	0.02	− 0.15	0.38*	1.00					
MUC2	0.09	− 0.12	0.003	0.08	1.00				
MPO	− 0.01	0.26*	0.37*	− 0.02	− 0.18*	1.00			
NEO	0.06	− 0.001	0.33*	0.05	− 0.11	0.35	1.00		
A1AT	− 0.07	− 0.01	− 0.06	0.21*	0.16	0.11	− 0.11	1.00	
EED score	− 0.06	0.17*	0.31*	0.08	− 0.08	0.80	0.42	0.53	1.00

*Statistically significant.

Significant values are in bold.

### Association of I-FABP and other variables with LAZ of the study participants

Multivariable linear regression analysis revealed that I-FABP is significantly and negatively associated with LAZ of the study participants (*β* =  − 0.337, 95% CI − 0.618, − 0.057; p-value = 0.019) after adjusting for all the variables. On the other hand, the mother’s education (*β* = 0.121, 95% CI 0.003, 0.240; p-value = 0.04) was found to be positively and significantly associated with LAZ of the study participants. In addition, LAZ of female participants (*β* = 0.773, 95% CI 0.122, 1.425; p-value = 0.020) were 0.773 units higher compared to male participants among the study groups. Table 4 illustrates the results of multivariable linear regression analysis.

**Table 4:** Multivariable linear regression analysis to see the association of other variables with LAZ.

Variables	Unadjusted β (95% CI)	p-value	Adjusted β (95% CI)	p-value
Age (month)	0.148 (0.008, 0.287)	0.038	0.032 (− 0.122, − 0.187)	0.678
Gender (female)	0.456 (− 0.148, 1.062)	0.138	0.773 (0.122, 1.425)	**0.020**
I-FABP (ng/mL)	− 0.368 (− 0.640, − 0.097)	0.008	− 0.337 (− 0.618, − 0.057)	**0.019**
TFF3 (ng/mL)	− 20.079 (− 48.820, 8.660)	0.169	− 6.993 (− 37.015, 23.027)	0.645
RBP4 (mg/L)	− 0.031 (− 0.074, 0.011)	0.149	− 0.030 (− 0.075, 0.014)	0.187
CRP (mg/L)	− 0.047 (− 0.085, − 0.009)	0.015	− 0.086 (− 0.180, 0.007)	0.071
Ferritin (ng/mL)	− 0.006 (− 0.027, 0.014)	0.545	− 0.019 (− 0.043, 0.004)	0.108
LCN 2 (µg/mL)	− 0.028 (− 0.045, − 0.010)	0.002	− 0.012 (− 0.036, − 0.011)	0.288
Lactoferrin (ng/mL)	0.003 (− 0.0004, 0.008)	0.08	0.003 (0.001, 0.008)	0.176
MUC2 (ng/mL)	0.01 (− 0.009, 0.030)	0.293	0.003 (− 0.013, 0.020)	0.668
MPO (ng/mL)	− 0.053 (− 0.087, − 0.020)	0.002	− 0.019 (− 0.064, 0.026)	0.403
NEO (nmol/L)	− 0.211 (− 0.323, − 0.098)	0.0001	− 0.052 (− 0.177, 0.072)	0.405
AAT (mg/g)	0.932 (0.119, 1.744)	0.025	0.401 (− 0.455, 1.258)	0.355
Mother height (cm)	0.113 (0.059, 0.167)	0.0001	0.054 (− 0.005, 0.113)	0.073
Mother education (years)	0.127 (0.003, 0.252)	0.045	0.121 (0.003, 0.240)	**0.044**

Significant values are in bold.

## Discussion

Our current study findings exhibited that TFF3 significantly and positively correlates with MPO which has emerged as a biomarker of EED in various studies^7,23^. TFF3 also positively correlates with the EED score in our study. Therefore, our results suggest that TFF3 might be a biomarker of EED in Bangladeshi children^16^. It was found to be correlated with intestinal inflammation as well as inflammation of solid tumours in various cancers including pancreatic cancer, gastric cancer, breast cancer, prostate cancer, and hepatocellular carcinoma^24^. It has also been reported as a biomarker of disease activity in patients with IBD, specially in Ulcerative Colitis^25^. Overall, our findings imply that TFF3 might be a promising biomarker and an indicator of intestinal inflammation and EED.

In this study, we also measured the levels of LCN2 in faecal samples. Correlation of LCN2 with other faecal biomarkers showed that LCN2 positively correlates with MPO and NEO; along with MPO, NEO has also been reported as a biomarker of gut inflammation and an indicator of EED in different studies^7,23,26–28^. This finding is in line with the result of another study, where LCN2 was found to be positively correlated with MPO^17^. Likewise, LCN2 has also been found to be positively correlated with the EED score in this study. Therefore, LCN2 might be a biomarker of intestinal inflammation and EED^17,29^. It has been reported as a potential biomarker of infection, inflammation, ischemia, and kidney damage^29^. It has also been found to be highly expressed in intestinal epithelial cells in IBD^29^. In addition, it has been reported as a sensitive and dynamic biomarker of intestinal inflammation^30^. Moreover, a transcriptomic study reported that LCN2 was highly expressed in duodenal biopsies of EED^31^. Another study also reported that expression of LCN2 gene was high in patients with severe acute malnutrition enteropathy^32^. Overall, our findings suggest that LCN2 might be an important biomarker of intestinal inflammation and an indicator of EED.

We also performed multivariable linear regression analysis, which revealed that I-FABP was significantly and negatively associated with LAZ of the study participants. This finding is supported by a study conducted in a rural area of Peru where LAZ was found to be strongly and inversely correlated with I-FABP among stunted children^33^. Another study also reported that I-FABP was negatively associated with LAZ and its concentration was higher in children who failed to respond to nutritional intervention. I-FABP was also found to be positively associated with pathogen burden in the same study^34^. Moreover, levels of plasma I-FABP were significantly higher in severely stunted children compared to healthy controls in our study. I-FABP is an intracellular epithelial protein located at the tips of villi of the small intestine that is released into the blood circulation following intestinal injury^1^. It has been reported as an indicator of the severity of intestinal damage in both adults and children, reflecting current gut injury^35,36^. Moreover, it has been found to be associated with worse stunting in children of Northeast Brazil upon principal component analysis^37^. Children living in resource-poor settings and unhygienic conditions are repeatedly exposed to a variety of intestinal pathogens^38–41^ whose ultimate consequence is intestinal inflammation and intestinal damage that causes linear growth faltering in LMICs^14^, thus it is inferable that higher levels of I-FABP represent the rapid turnover of intestinal mucosa and ongoing damage of intestinal epithelial cells. Moreover, it has also been reported as a future risk of growth faltering as well as found to be associated with stunting^42^. Therefore, our study findings imply that I-FABP is associated with linear growth and also suggest it as a possible biomarker of stunting and EED of Bangladeshi children. Multivariable linear regression also reported that mother’s education was positively and significantly associated with LAZ of the study participants in this study. This result suggests that mother’s education has a positive influence on the nutritional status of children. This finding is consistent with the results of other studies where mothers with no/primary education have higher odds of having stunted children compared to mothers with secondary/higher education^43,44^. In our study, LAZ for female participants were found to be higher compared to male participants. Male gender was also found to be associated with higher probability of being stunted compared to female^43^, which is consistent with our result. Another study also reported that male children are more prone to become stunted compared to their female counterparts and implied that boys are more vulnerable to health inequalities compared to girls in under 5 age group^45^. Moreover, other variables including TFF3, RBP4, CRP, Ferritin, LCN2, Lactoferrin, MUC2, MPO, NEO, A1AT, and mother height were found to be non-significant after adjusting in the multivariable model; this may be due to small sample size. These findings are in line with the results of another study conducted in the same setting^46^, where MPO, NEO, A1AT, and CRP were not found to be associated with LAZ of the study participants.

There were some limitations in our study. Firstly, we could not see the expression of TFF3 and LCN2 genes due to invasive collection process of intestinal cells. Secondly, we included single time point data for all the variables, which reduced our ability to capture the potential relationship among the biomarkers as well as the association of different variables with LAZ of the study participants. Furthermore, due to lack of cut-off value for EED score, we were unable to validate the specificity and sensitivity of the TFF3 and LCN2 by using ROC curve. However, this was the first study to investigate the relationship of TFF3 with biomarkers of EED as well as the association of I-FABP with linear growth in Bangladeshi children.

In conclusion, our results suggest that TFF3 and LCN2 might be promising biomarkers to diagnose gut inflammation and EED. Moreover, I-FABP is negatively associated with linear growth. However, maternal education and female gender are positively associated with linear growth in Bangladeshi children. Though our study implies that these biomolecules might be biomarkers of EED, there is no cut-off value for these biomolecules and EED score. So, a larger longitudinal study is needed to determine the cut-off value for these biomolecules where we can compare these biomolecules and EED score with histopathological score of EED.

## Data Availability

The data set that was created during the study is not publicly available due to the restrictions of the funder. However, suggestions for data analysis can be made to the corresponding author.
